# Spin separation based on-chip optical polarimeter via inverse design

**DOI:** 10.1515/nanoph-2021-0455

**Published:** 2021-10-14

**Authors:** Changyu Zhou, Youpeng Xie, Jianxin Ren, Zepeng Wei, Luping Du, Qiang Zhang, Zhenwei Xie, Bo Liu, Ting Lei, Xiaocong Yuan

**Affiliations:** Nanophotonics Research Centre, Institute of Microscale Optoelectronics, Shenzhen University, Shenzhen, 518060, China; Institute of Optics and Electronics, Nanjing University of Information Science and Technology, Nanjing 210044, China

**Keywords:** inverse design, polarimetry, Stokes vector direct detection

## Abstract

Polarimetry has been demonstrated essential in various disciplines, such as optical communications, imaging, and astronomy. On-chip nanostructures for polarization measurements are most expected to replace the conventional bulk elements, and hence minimize the polarimeter for integrated applications. Some on-chip nanophotonic polarimeter via polarization detection has been implemented, in which the separation of two spin polarized states is needed. However, due to the relatively low coupling efficiency or complicated photonic silicon circuits, on-chip polarimetry using a single device still remains challenging. Here, we introduce and investigate an on-chip polarimeter with nanostructures using the inverse design method. The developed device shows the ability to detect the four polarization components of light, two of which are the spin polarizations, and the other two are the linear polarizations. The retrieved Stokes parameters with experimentally tested data are in close agreement with the numerical results. We also show the proof of concept demonstration for high-speed Stokes vector optical signals detection. In the high-speed communication experiment with data rate up to 16 GBd, the detected optical signals via polarization measurements at multiple wavelengths in the C-band were recovered with the bit error rate below the 20% forward error correction threshold. The proposed on-chip polarimeter shows promising performance both in Stokes polarimetry and high-speed optical communication applications.

## 1 Introduction

The state of polarization (SOP) of light plays the crucial role in light–matter interactions [1, 2]. In the reverse process, the SOP extracted from light–matter interactions using a polarimeter has broad applications in imaging [3], [4], [5], optical communication [6], [7], [8] and astronomy [9]. Traditional polarimeters are typically the combination of linear polarizer, quarter wave plate, and other optical elements, which are bulky and expensive for mass production and integration applications. Recent advances of nanophotonic technology have paved the way to shrink the polarimeter for the sake of integration. Some polarimetric strategies based on metasurface have been demonstrated with high efficiency for free-space applications [4, 10], [11], [12], [13]. To further miniaturize the polarimeter for on-chip applications, one possible method is using the plasmonic resonant nanoantennas [14], but with large optical loss of metal. The other integrated nanophotonic devices using the silicon photonics technology have also been implemented [15], [16], [17], [18], [19]. However, most of them were accompanied with complex optical waveguide circuits which require high precision fabrication of photonic silicon devices. In the majority of above realizations for on-chip applications, an essential method is based on the separation of two orthogonal spin polarization states. As a manifestation of photonic spin-Hall effect [20], [21], [22], the spin-momentum locking, in which the transverse spin is locked with the propagation direction of light, has been demonstrated in various platform, including in evanescent wave [23, 24] and light scattering process [25]. Intrinsic spin–orbit coupling of light has been proposed to design the on-chip polarimeters using a single nanostructure [26, 27], but typically with relatively low coupling efficiency due to the weak spin–orbit coupling [26]. While the inverse design method provides a solution to balance the coupling efficiency and the complexity of device for on-chip polarimetry.

Inverse design has been introduced to photonics and photonic device design [28], [29], [30], [31], [32], [33], [34], including on-chip optical emitter [31], spin Hall device [30] and wavelength/mode demultiplexer [32], [33], [34], with the advantage of exploring optimal design of the devices. This provides an opportunity to achieve an optimal performance for polarimetry on an integrated circuit. In reference [30], the two orthogonal spin polarization states are guided to propagate along opposite direction in waveguide, which is insufficient to achieve the polarimetry. To retrieve the polarization states of light, another two linear-polarization components should also be considered [35]. In this work, we demonstrate an integrated four-channels polarimeter on a standard silicon on insulator (SOI) substrate using the inverse design method. The developed device shows the ability to detect the four polarization components of light, two of which are the left circular (LCP) and right circular polarization (RCP) components, and the other two are the 0° and 135° linear polarization (LP) components. We implemented the measurements of arbitrary SOP on the Poincaré sphere. The retrieved Stokes parameters with experimentally tested data are consistent with the numerical results. In addition, the proposed device also has ultrafast time response and short data delay among different channels, which are superior properties to achieve the phase demodulation of the coherent optical data transmission. We demonstrate the high-speed coherent optical demodulation with the 16-GBd quadrature phase-shift keying (QPSK), eight phase-shift keying (8PSK) and 16-ary quadrature amplitude modulation (16QAM) signals by Stokes vector direct detection. We tested the QPSK, 8PSK, and 16QAM signals with different input optical power and multiple wavelengths in the C-band, and the measured bit error rate (BER) demonstrated to be below the 20% forward error correction (FEC) threshold. This on-chip polarimeter is compatible for complementary metal–oxide–semiconductor mass production and integration with the microelectronic devices, and provides a reliable method for Stokes analyzing in optical communication systems with ultracompact size.

## 2 Device design

**Figure 1:**
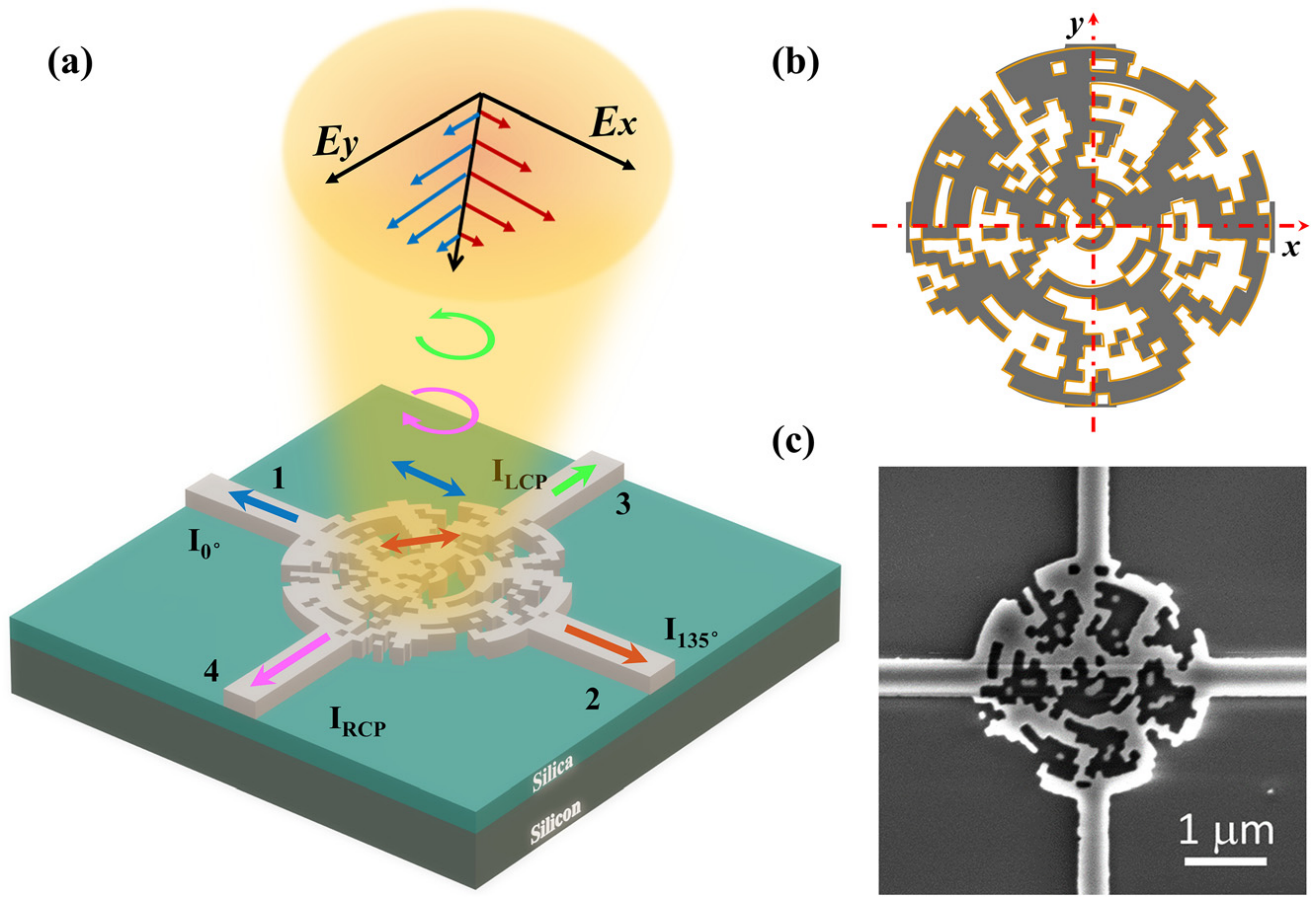
Structure design of the on-chip polarimeter. (a) Schematic of the on-chip polarimeter. (b) The design area is a circular area with a diameter of 3 μm composed of 660 pixels, each of which is a nano arc area that can be filled either with silicon or air. (c) SEM image of the fabricated device. The scale bar is 1 μm.

The proposed on-chip polarimeter consists of four single-mode silicon waveguides coupled to a circle-like device area, with the functionality to detect the polarization of the incoming light through the intensity measurements at the outputs of the waveguides (Figure 1). The SOP of the incident light can be retrieved by measuring the intensities of the RCP, LCP, 0° and 135° LP components, and these components are directly sorted out to different waveguide ports. Eventually, the incident SOP can be decided from these intensities, as well as the Stoke parameters.

We adopt the inverse design method to optimize the device. The device is designed on a standard silicon-on insulator (SOI) wafer, in which the top silicon layer is 220 nm thick. The device area is circle-like with a diameter of 3 μm and divided into 660 circular arc-shape pixels, and the size of each pixel is around 100 nm. We choose the global optimization that combines the annealing algorithm with three-dimensional finite-difference time-domain (FDTD) simulations to optimize the material distribution of the device. The figure of merit (FOM), a key parameter to characterize the process in the optimization, is defined by the weighting value between the average and the standard deviation of the coupling efficiencies in the four channels for the four input polarizations (LCP and RCP, 0° and 135° LP). In the annealing algorithm, the initial temperature is set as large as possible to ensure a large search space.

The device consists of a random mixture of silicon pixels and air pixels. During the optimization in the FDTD simulation, the coupling efficiencies are recorded from the corresponding channels for vertically incoming light of different SOPs. The initial FOM is calculated following the first simulation with a random material distribution. By flipping the material component (from silicon to air or vice versa) of a random pixel, we calculate the FOM again following the FDTD simulation. If the FOM value is larger than the former one, its new value is recorded and the material component flip is accepted. Otherwise the new value and the flip are accepted by the probability of exp [(*γ*_*n*_ − *γ*_*n*−1_)/*T*]. where *T* is the annealing temperature, *γ*_*n*_ and *γ*_*n*−1_ are the FOM values in the *n*th and (*n*−1)-th simulation, respectively. Each pixel is flipped equally once per iteration, and the annealing temperature is then reduced according to *T* → 0.9*T* in the next iteration, where the factor 0.9 is the cooling rate. This process continues until the FOM value reaches its maximum or the iteration number exceeds the default value.

**Figure 2:**
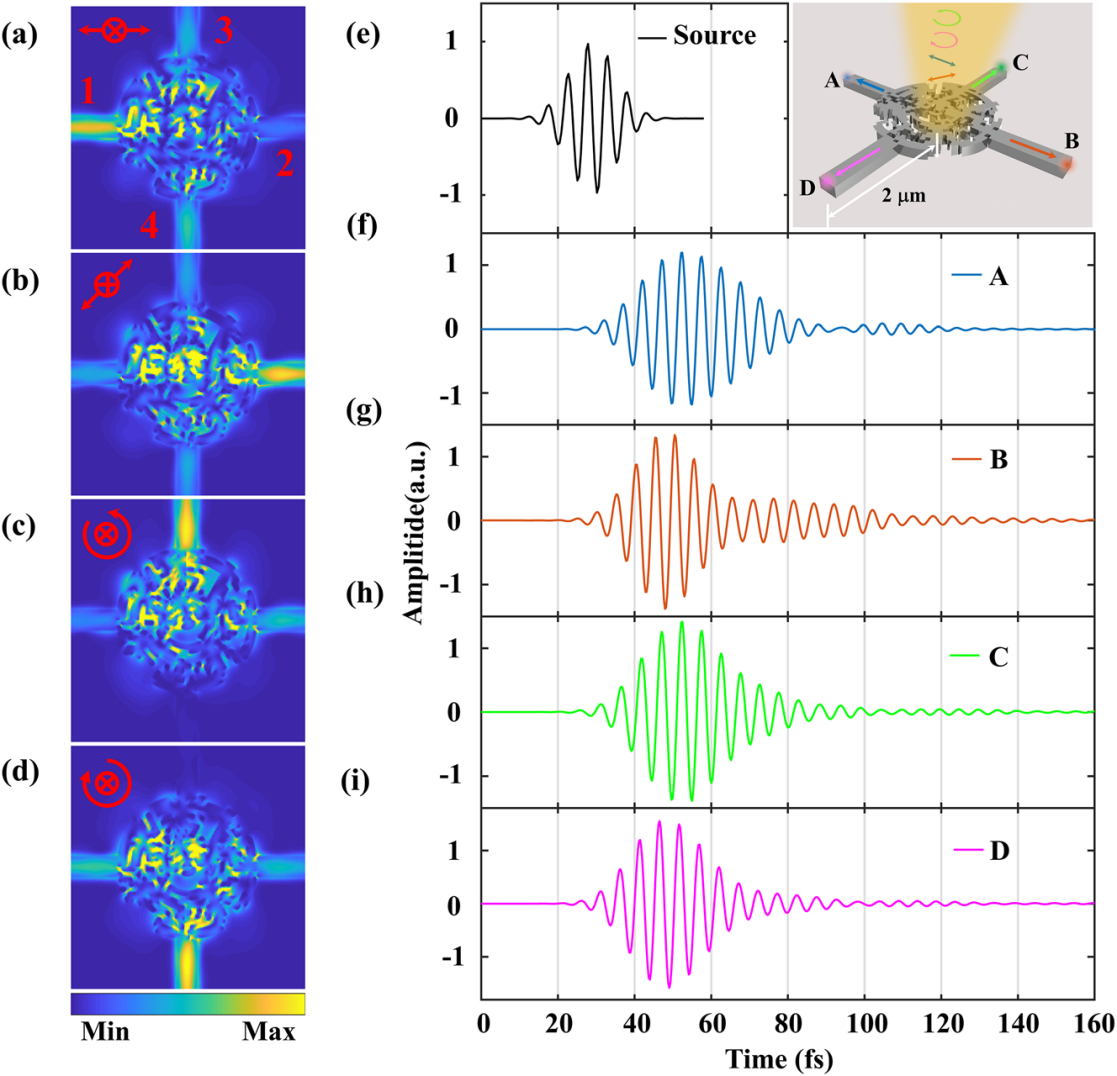
Simulation results of the device. (a)–(d) Electric field intensity profiles with the vertically incident beam of (a) 0°, (b) 135°, (c) LCP, and (d) RCP states. (e) Time-domain response of the incident 20-fs pulse. The pulse traces measured at points A, B, C, and D indicated in the right inserts are simulated under input 0° LP, 135° LP, LCP, and RCP light, respectively (marked by different colors), and these points are located at the same distance of 2 μm from the circle-like device center. (f)–(i) Pulse traces at the output of the Si waveguide for (f) 0° LP, (g) 135° LP, (h) LCP, and (i) RCP components.

The FDTD simulation is implemented to investigate the performances of the optimized device with vertical illumination of two circular states (LCP and RCP) and two LP states (0°, 135°). The simulation results are shown in Figure 2a–d. Light is scattered into desired channels by hundreds of silicon nano elements inside the inverse designed circle-like device. The 0° LP light couples into the left channel (port 1) and the 135° LP couples into the right channel (port 2), while the LCP couples into the upper channel (port 3) and RCP couples into the lower channel (port 4). On the other hand, for the sake of high-speed signals detection with the developed device, the time-domain simulation is also implemented (Figure 2). The simulation results of the device show the pulse duration is increased to 40 fs. Although the pulse broadening is significant compared to the 20-fs source pulse, the pulse arrival time is not affected and the broadening is negligible for the parameters of modern optical communication systems. Therefore, it is possible to achieve the high-speed simultaneous measurements of the Stokes parameters using this device.

## 3 On-chip Stokes polarimetry

**Figure 3:**
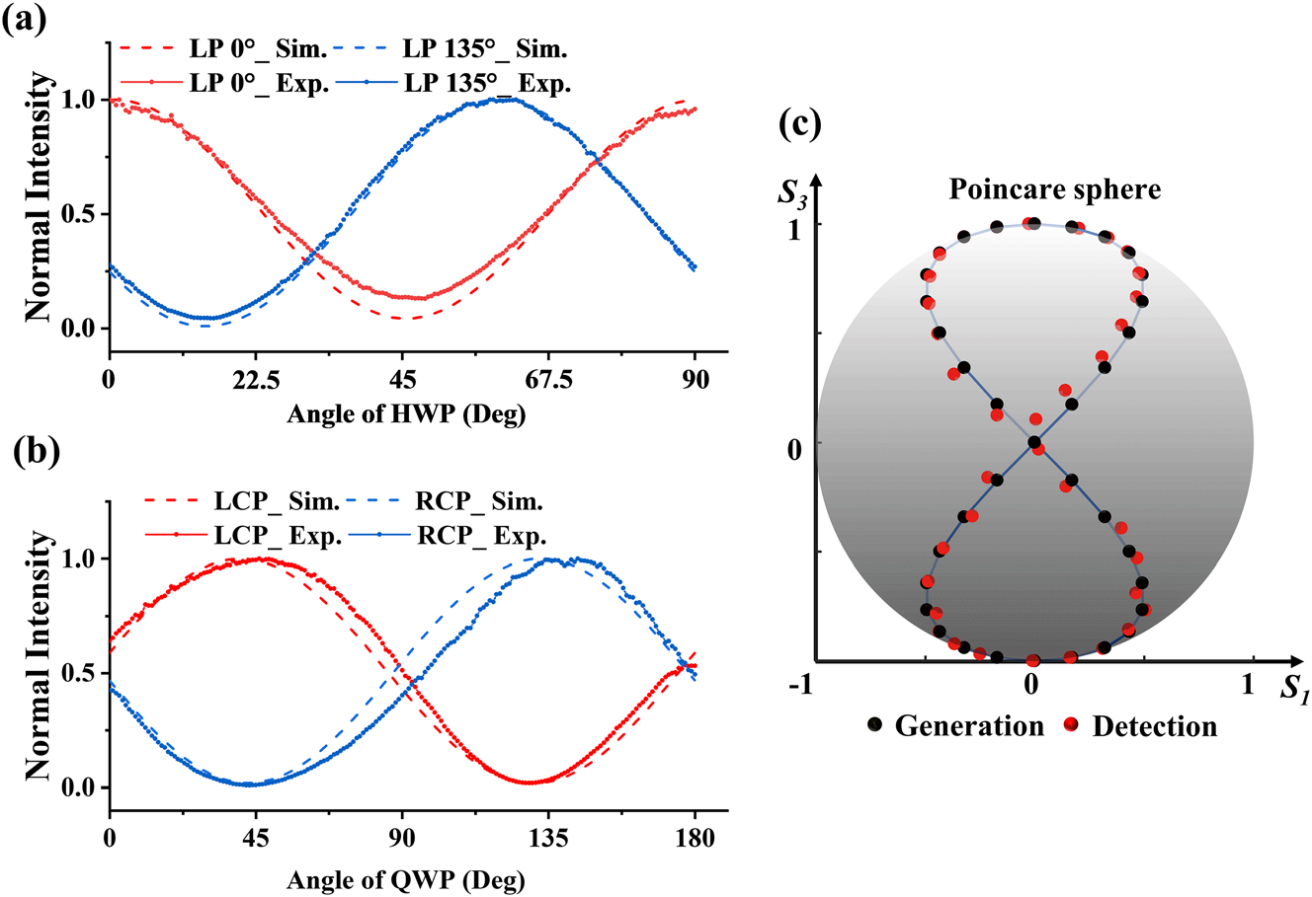
Device characterization for Stokes polarimetry. (a) Simulated and experimentally detected intensities with varying HWP for 0° and 135° LP components. (b) Simulated and experimentally detected intensities with varying QWP angles for LCP and RCP components. (c) Retrieved 36-sets Stokes parameters on Poincaré sphere with varying QWP from 0° to 180° and fixing the HWP angle at 0°.

We have experimentally characterized the performance of the device for Stokes polarimetry (see supplementary materials for details). The measured optical response curves of port 1 (0°) and port 2 (135°) with the rotation angle of HWP from 0° to 90° are shown in Figure 3a. While the measured optical response curves of the port 3 (LCP) and port 4 (RCP) with rotation angle of QWP from 0° to 180° are shown in Figure 3b. The solid line represents the experimental results and the dashed line represents the simulation results. The measured curves of the four ports are in close agreement with the simulation curves. It should be noticed that both the simulated and experimentally tested peak values of the response curves for 135° LP in Figure 3a are at angle 60° of HWP (blue curves) and the results deviate from the desired angle of HWP at 67.5°. This deviation is due to the setting of FOM during the inverse optimization, the FOM in the optimization process ensures the maximal efficiencies and minimal efficiency differences of four ports rather than the response curve to desired polarization (135° linearly polarized light). However, such a deviation will not influence the restored results of Stokes parameters.

The ratio of the maximum optical power and the minimum optical power of a port is defined as the extinction ratio. The experimentally measured extinction ratios for the 0° and 135° LP channels are larger than 9 dB (port 1) and 13 dB (port 2), respectively, which are smaller than the simulation results of 13 dB at port 1 and 20 dB at port 2. The intensity extinction ratios were measured to be 17 dB for the LCP channel (port 3) and 20 dB for the RCP channel (port 4), and are slightly higher than the numerically calculated results of 16 dB at port 3 and 17 dB at port 4 (see more experimentally tested efficiencies in Supplementary materials). The numerically calculated coupling efficiency, defined as the ratio of the power at the corresponding output port to the input power, is approximately 10% for each port. The Stokes parameters can be restored from the detected *I*_0_, *I*_135_, *I*_LCP_, and *I*_RCP_ intensities through the formula [35]

(1) S=[S0S1S2S3]=[|Ex|2+|Ey|2|Ex|2−|Ey|22Re{Ex⋅Ey∗}2Im{Ex⋅Ey∗}]=M[ILCPI0I135IRCP] ,

where *E*_*x*_ and *E*_*y*_ are *x*- and *y*-polarized complex electric fields components, the superscript ∗ denotes the complex conjugate operation, *I* = [ *I*_LCP_ *I*_0_ *I*_135_ *I*_RCP_ ]^*T*^ is the intensities vector of the respective polarization components, and *M* represents a 4 ⨯ 4 retrieval matrix, which includes the necessary information related to coupling and propagation loss and the corrections to other effect caused by the silicon waveguide, fiber, and detector. Figure 3c shows the retrieved results of 36 sets of Stokes parameters with the angle of QWP from 0° to 180°. The polarizations of light will vary between linear polarization and elliptical polarization, and the retrieved Stokes parameters with experimentally tested data are in close agreement with the numerical results.

## 4 High-speed Stoke vector direct detection

**Figure 4:**
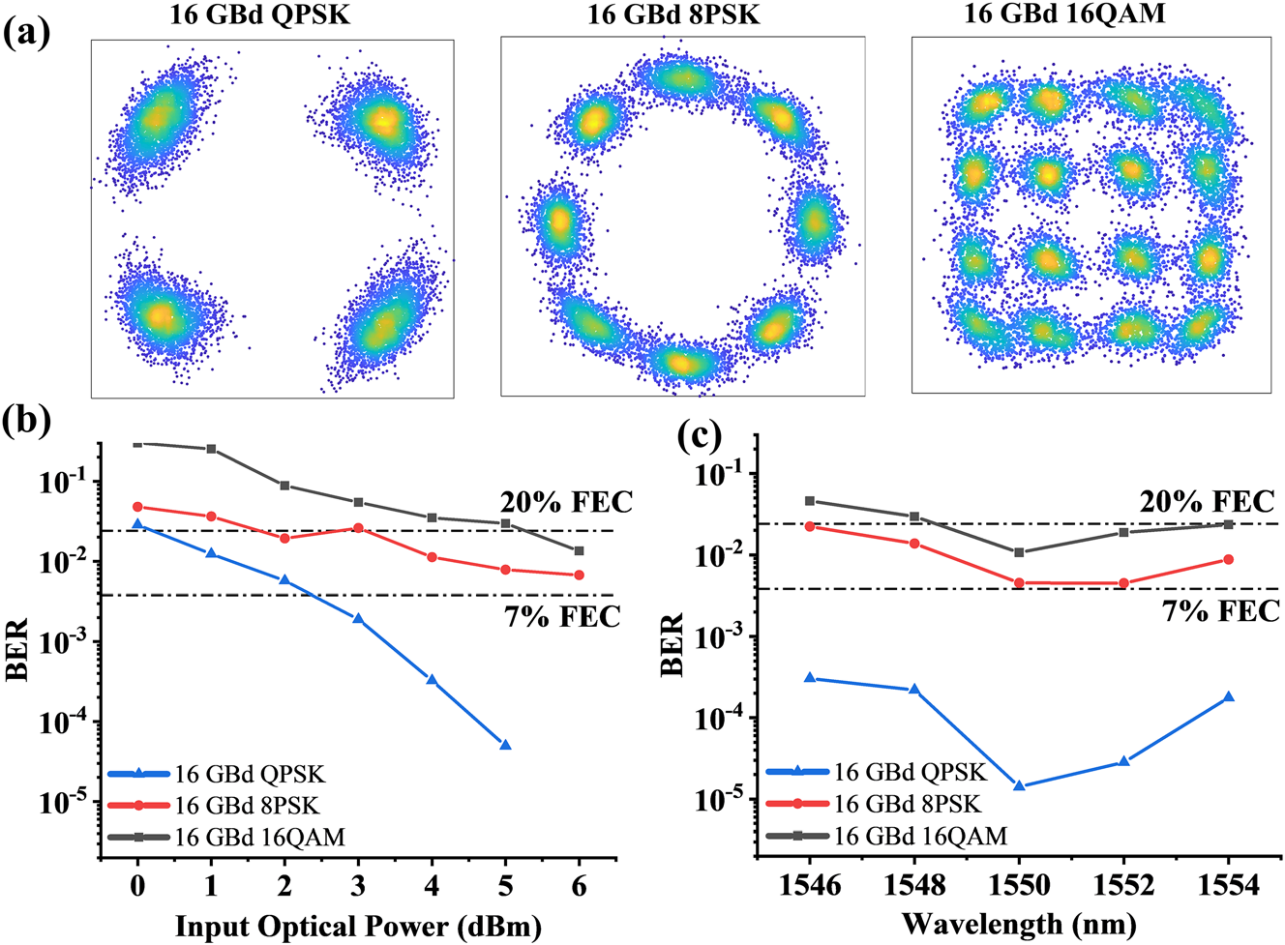
High-speed Stokes vector direct detection experiment. (a) Recovered constellation diagrams for 16-GBd QPSK, 8PSK, and 16QAM modulation formats using experimentally tested data (tested wavelength: 1550 nm, input power: 6 dBm). (b) BER curves of different input optical power (tested wavelength: 1550 nm). (c) BER curves of different tested wavelengths (input power: 6 dBm).

We have also experimentally tested the device for high-speed optical communication using the Stokes vector direct detection. The dual polarization modulator generates the *x*-polarization high-speed optical signals and the *y*-polarization phase reference. The tested coherent optical signals include the QPSK, 8PSK, and 16QAM formats, which can be directly detected by our device. The Stokes parameters of the original optical signals can be recovered from the sampled waveforms by the transmission matrix as shown in Eq. (1). By using the *y*-polarization component as the phase reference, the QPSK/8PSK/16QAM signals on the *x*-polarization component can be demodulated and equalized with a least mean squares equalizer. The information of complex electric field of these input signals can then be obtained from the measured Stokes parameters using Eq. (S4) in Supplementary materials. The retrieved constellation diagrams for different formats are shown in Figure 4a. The retrieved symbols are mapped back to the binary sequences using the Gray code method. The BER can be calculated by comparing the retrieved binary sequences with the sending sequences. We have measured the BER with different input powers from 0 to 6 dBm, and the BER curves of 16-GBd QPSK/8PSK/16QAM signals are shown in Figure 4b. The BER for 16-GBd QPSK signals is 0 when the input optical power exceeds 5 dBm. While the BERs for 16-GBd 8PSK and 16QAM signals are lower than 20% FEC threshold when the incident optical power exceeds 6 dBm. The BER can be further reduced by increasing the input power, which is limited by the system loss in our experimental setup (Figure S2). We have also measured the BER curves of QPSK/8PSK/16QAM signals with the wavelengths from 1546 to 1554 nm at 6 dBm of the input power, the tested wavelength interval is 2 nm, and the results are shown in Figure 4c. The BERs for the 16-GBd QPSK signals are below the 7% FEC threshold in the whole tested spectral range. Similarly, the BERs for 16-GBd 8PSK and 16QAM signals are lower than the 20% FEC threshold in some wavelength range. This device shows better performance for demodulation of QPSK signals since the QPSK signals have simpler polarizations distribution.

## 5 Conclusions

We have designed and demonstrated a four-ports polarimeter with nano-sized pixels on a standard SOI substrate using the inverse design method. The developed device shows the ability to detected the LCP, RCP, 0° and 135° LP components of the input light. By measuring the intensities of these components, we obtain the Stokes parameters of the input light beam using a transmission matrix. We demonstrated the detection of arbitrary polarization states using this device. The retrieved Stokes parameters with experimentally tested data are consistent with the numerical results. In addition, the proposed device also has extremely short time response for all channels, and we also demonstrated the high-speed coherent optical demodulation with the 16-GBd QPSK, 8PSK, and 16QAM signals by Stokes vector direct detection. The measured BER curves are demonstrated to be below the 20% FEC threshold. This inverse designed on-chip polarimeter has the advantages of ultracompact size and CMOS compatibility, which can be used for practical applications including in polarization detection, sensing, imaging, and optical communication.

## Supplementary Material

Supplementary Material Details
