# Supplementary material for: Spin separation based on-chip optical polarimeter via inverse design
Source: Nanophotonics. 2021 Oct 14;11(4):813–9. doi: 10.1515/nanoph-2021-0455 (PMC11501590; doi:10.1515/nanoph-2021-0455)
Supplement: Supplementary file 1 — Supplementary Material Details [file j_nanoph-2021-0455_suppl.docx]

Supplementary materials

**Spin separation based on-chip optical polarimeter via inverse design**

Changyu Zhou^1, #^, Youpeng Xie^1, #^, Jianxin Ren^2^, Zepeng Wei^1^, Luping Du^1^, Qiang Zhang^1^, Zhenwei Xie^1^, Bo Liu^2, *^, Ting Lei^1,^ *^*^*, Xiaocong Yuan^1,^ *^*^*

*^1^Nanophotonics Research Centre, Institute of Microscale Optoelectronics, Shenzhen University, Shenzhen, 518060, China*

*^2^Institute of Optics and Electronics, Nanjing University of Information Science and Technology, Nanjing 210044, China*

*^#^These authors contributed equally to this work.*

**Correspondence: bo@nuist.edu.cn, leiting@szu.edu.cn and* [*xcyuan@szu.edu.cn*](mailto:xcyuan@szu.edu.cn)

**S1. Performances of the device**

Following the inverse optimization process, we implemented the finite-difference time-domain (FDTD) simulation to obtain the coupling efficiencies of the device, which are shown as follows:


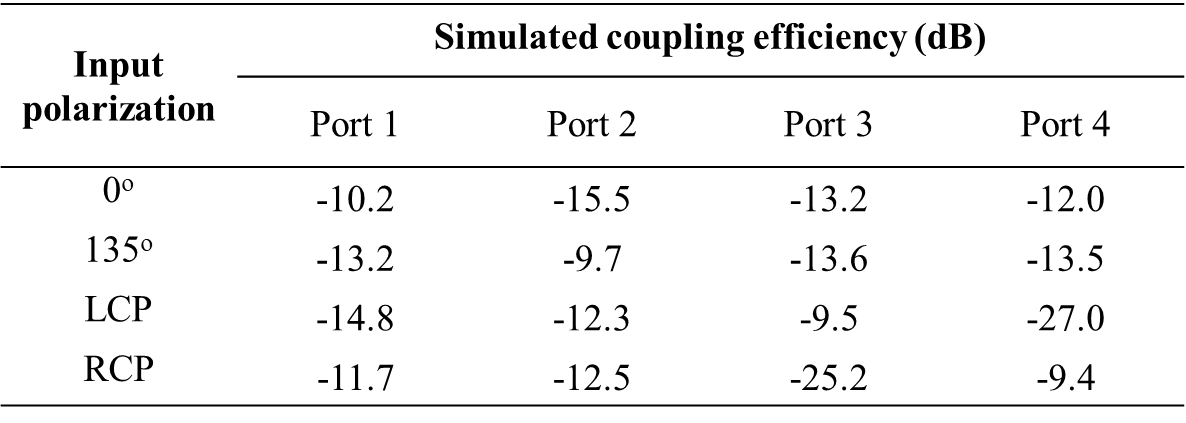


Table S1. FDTD simulation for coupling efficiencies of the device.

The coupling efficiency for each port corresponds to optimized desired polarization (e.g. port 1 corresponds to 0° input polarized light) is approximately -10dB (10%). While the direct detection of coupling efficiency is difficult in our experimental system because the transmission loss in Si waveguide and the coupling loss between the Si waveguide and lensed fiber are unknown in our present design. We instead tested the device and obtained the total efficiencies that include these losses (Table S2).


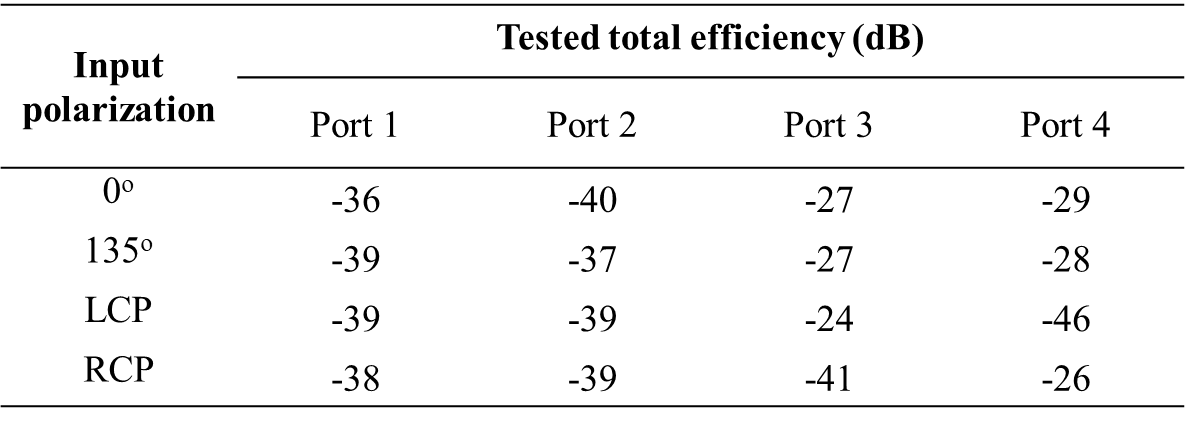


Table S2. Experimentally measured total efficiencies of the device.

**S2. Experimental characterization for Stokes polarimetry**


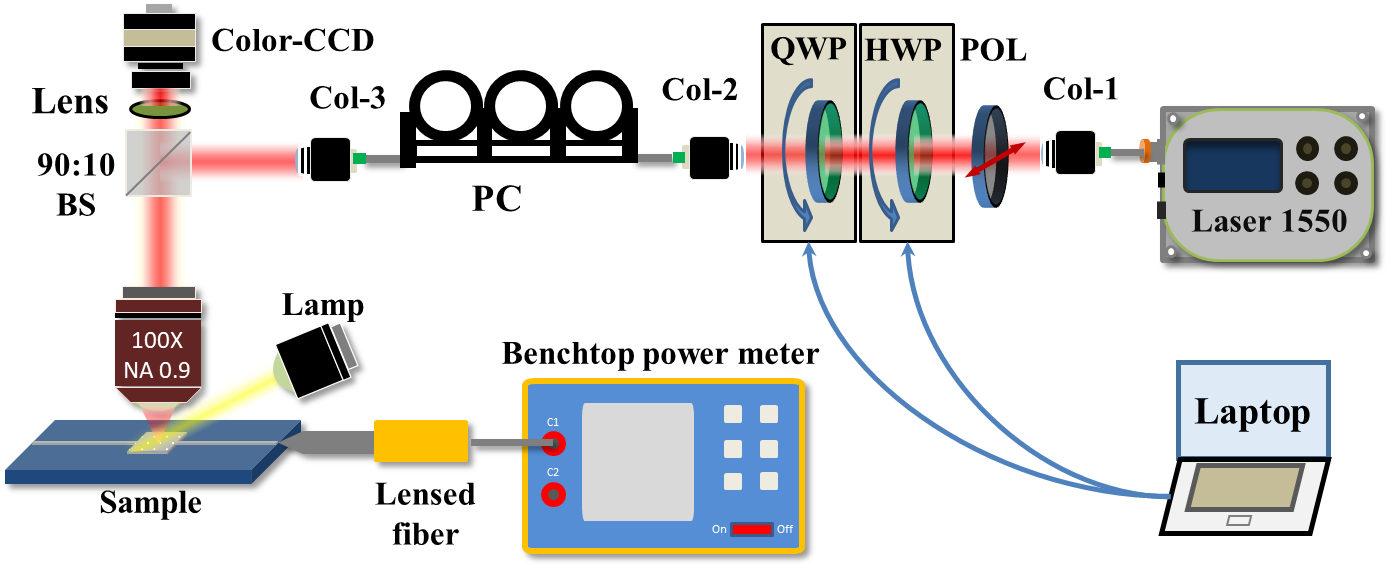


Figure S1. Experimental setup for Stokes polarimetry. PC: polarization controller; HWP: half-wave plate; QWP: quarter-wave plate; POL: polarizer; PD: photodetector; Col: collimator; BS: beam splitter.

Figure S1 shows the experimental setup. The combination of a half-wave plate (HWP) and a quarter-wave plate (QWP) were used to modulate the polarizations of light. By precisely controlling the rotations of HWP and QWP, one can obtain arbitrary desired polarization. Two identical collimators were used to couple and collimate the beam so as to decrease the beam wander when rotating the rotation mounts. The modulated light was coupled into a single-mode fiber and a polarization controller was adjusted to keep the polarization state of the output beam invariant. The last collimated beam was reflected by a 90:10 beam splitter (BS). An objective lens (NA=0.9, 100⨯) was fixed on a one-dimensional precision electric translation stage that can move vertically. Light focused by the objective lens was incident on the device after adjusting the positions of the incident focused beam and the device.

The device was fixed on a two-dimensional motorized translation stage (ASI, MS2000). A lamp was used to shine on the device. A lens was placed upon the BS to compensate the focal length of visible light for imaging. A colored charge coupled device was used to observe the position of the device in real time. A lensed fiber was fixed on a three-dimensional nano-translation stage to couple the light output from each waveguide. The optical power of light at the output from the lensed fiber was recorded with a benchtop power meter (EXFO, IQS-1700 with IQS-600 integrated qualification system).

**S3. Retrieval methods for Stokes vector**

The retrieval methods of Stokes vector are shown as follows. The transmitted optical signals can be represented using the Jones vector

$E=[E_{x},E_{y}]^{\mathrm{T}},$ (S1)

where *E_x_* and *E_y_* are the transmitted *x*-polarization and *y*-polarization complex fields, respectively. ‘T’ represents the transpose of the matrix. The relation between Stokes vector ***S*** and complex fields ***E*** can be expressed as

$S=\left[\begin{matrix} S_{0} \\ S_{1} \\ S_{2} \\ S_{3} \end{matrix}\right]=\left[\begin{matrix} \left|E_{x}\right|^{2}+\left|E_{y}\right|^{2} \\ \left|E_{x}\right|^{2}-\left|E_{y}\right|^{2} \\ 2\mathrm{Re}\left\{E_{x}\cdot E_{y}^{*}\right\} \\ 2\mathrm{Im}\left\{E_{x}\cdot E_{y}^{*}\right\} \end{matrix}\right],$ (S2)

where S_0_, S_1_, S_2_, and S_3_ are the Stokes vector components. The Stokes vector can be recovered by a transmission matrix as follows [1]

$\left[\begin{matrix} S_{0} \\ S_{1} \\ S_{2} \\ S_{3} \end{matrix}\right]=MI=\left[\begin{matrix} m_{11} & m_{12} & m_{13} & m_{14} \\ m_{21} & m_{22} & m_{23} & m_{24} \\ m_{31} & m_{32} & m_{33} & m_{34} \\ m_{41} & m_{42} & m_{43} & m_{44} \end{matrix}\right]\left[\begin{matrix} I_{L} \\ I_{0^{^{\circ}}} \\ I_{135^{^{\circ}}} \\ I_{R} \end{matrix}\right],$ (S3)

where *M* is the transmission matrix, *I*_L_, *I*_0_^o^, *I*_135_^o^, and *I*_R_ are optical powers of LCP, 0° LP, 135° LP and RCP components. The transmission matrix can be used to compensate the efficiency difference and coupling loss of each port of the device. It can also compensate for polarization rotation and instability caused by bending and jitters in a SMF. On the other hand, the Jones vector describing the complex electric field of the input signal can be obtained from the measured Stokes vector as

$E=\left[\begin{matrix} E_{x} \\ E_{y} \end{matrix}\right]=C\left[\begin{matrix} \sqrt{1/2(1+S_{1})} \\ \sqrt{1/2(1-S_{1})}\exp[i\arctan(S_{3}/S_{2})] \end{matrix}\right]\text{，}$ (S4)

where *C* is a constant. Using the least square formula of the matrix to calculate the transmission matrix with the measured intensities of the four ports, the Stokes parameters can be retrieved, and the results are shown in figure 3(c) of the main text.

**S4. High-speed signals detection setup**

Figure S2 shows the setup for high-speed Stokes vector direct detection. The high-speed optical signals were generated by a dual polarization optical transmitter (ID photonics, OMFT). The optical transmitter was driven by an arbitrary waveform generator (AWG, Keysight M8195A) with four channels. The generated digital modulation signals include quadrature phase-shift keying (QPSK), eight phase-shift keying (8PSK) and 16-ary quadrature amplitude modulation (16QAM) signals in *x*-polarization and modified signals that act as a phase reference in *y*-polarization. The *y*-polarization component has regular phases of π/4 or 5π/4. The digital modulation signals pass through a rising cosine pulse shaping filter that has a rolling coefficient of 0.35 so as to reduce the crosstalk between adjacent symbols. The filtered digital signals were loaded on the corresponding channel of the arbitrary waveform generator to drive the optical transmitter. The modulated light carrying high-speed signals was transmitted through a standard single mode fiber (SMF). The modulated optical signals were amplified using an erbium-doped fiber amplifier (EDFA) and filtered using a tunable optical filter (Santec OTF-350). We used a variable optical attenuator (VOA) to adjust the optical output power. The light carrying high-speed signals was reflected by the 90:10 BS and focused by the objective lens, and then was incident on the four-ports device after adjusting the positions of the incident light focus and device. The optical signals were demodulated by the device. The output lights from the four ports of the device are 0° LP, 135° LP, LCP and RCP components of incident light carrying high-speed signals.


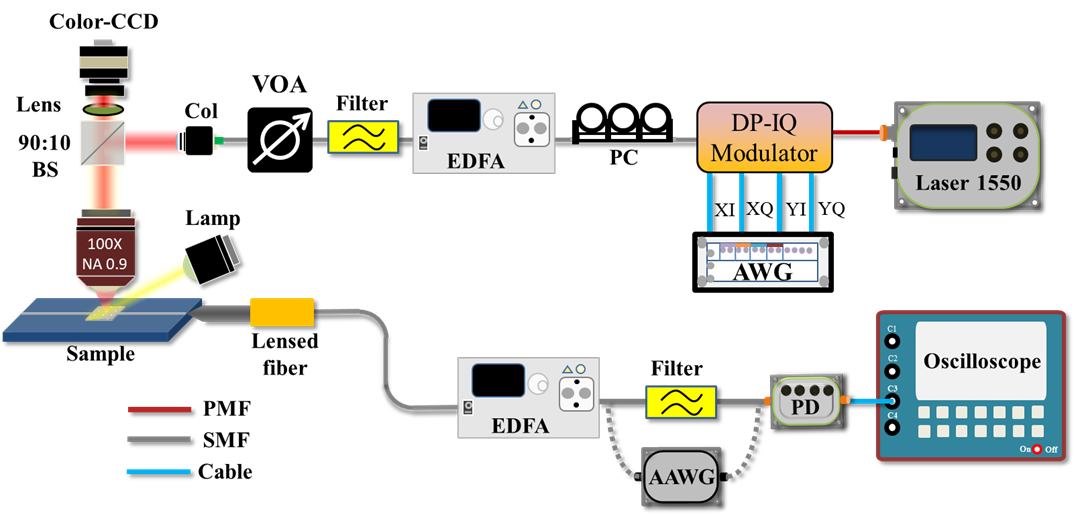


Figure S2. Setup for Stokes vector direct detection experiment. PMF: polarization-maintaining fiber; DP-IQ modulator: dual-polarization in-phase quadrature modulator; AWG: arbitrary waveform generator; SMF: single-mode fiber; PC: polarization controller; EDFA: erbium-doped fiber amplifier; OTF: optical tunable filter; VOA: variable optical attenuator; POL: polarizer; Col: collimator; AAWG: athermal arrayed waveguide grating; PD: photodetector; BS: beam splitter.

At the receiving terminal, the high-speed optical signals were amplified using a small signal EDFA and followed by a filter (for a single wavelength measurements) or an athermal arrayed waveguide grating (C band, 50-GHz channel spacing) module for multi-wavelengths test. A commercial photodetector (Finisar MPRV1331A) was used to detect high-speed intensity changes. The changes in the light intensities of the different signals in each port were converted into electrical signals by the photodetector. We used the oscilloscope (Agilent DSA-X93204A) to record the electric signals, which can be used to numerically restore the measured retrieval matrix *M* and the phase of the signals with Eq. S3 and Eq. S4. We tested the random 16-GBd QPSK/8PSK/16QAM signals of different power or different wavelengths and the bit error rate was then calculated using the detected signals (Figure 4).

**S3. Fabrication methods**

The inverse designed device was fabricated on a standard silicon on insulator (SOI) wafer with a 220-nm-thick device layer and a 3-μm-thick buried oxide layer. The pattern is defined with e-beam photolithography using the Raith EBPG 5200. The photoresist is PMMA with a thickness of 160 nm, having high etching selectivity to silicon.

[1] Che, D.; Shieh, W., Polarization Demultiplexing for Stokes Vector Direct Detection. *Journal of Lightwave Technology,* *34* (2), 754-760, 2016.
